# Disrupted extracellular matrix and cell cycle genes in autism-associated Shank3 deficiency are targeted by lithium

**DOI:** 10.1038/s41380-023-02362-y

**Published:** 2023-12-20

**Authors:** Valentin Ioannidis, Rakshita Pandey, Helen Friedericke Bauer, Michael Schön, Jürgen Bockmann, Tobias M. Boeckers, Anne-Kathrin Lutz

**Affiliations:** 1 Institute for Anatomy and Cell Biology, Ulm University, 89081 Ulm, Germany; 2 International Graduate School in Molecular Medicine Ulm, Ulm University, Ulm, Germany; 3 German Center for Neurodegenerative Diseases (DZNE), Ulm site, 89081 Ulm, Germany

**Keywords:** Autism spectrum disorders, Neuroscience

## Abstract

The Shank3 gene encodes the major postsynaptic scaffolding protein SHANK3. Its mutation causes a syndromic form of autism spectrum disorder (ASD): Phelan-McDermid Syndrome (PMDS). It is characterized by global developmental delay, intellectual disorders (ID), ASD behavior, affective symptoms, as well as extra-cerebral symptoms. Although Shank3 deficiency causes a variety of molecular alterations, they do not suffice to explain all clinical aspects of this heterogenic syndrome. Since global gene expression alterations in Shank3 deficiency remain inadequately studied, we explored the transcriptome in vitro in primary hippocampal cells from Shank3∆11(−/−) mice, under control and lithium (Li) treatment conditions, and confirmed the findings in vivo. The Shank3∆11(−/−) genotype affected the overall transcriptome. Remarkably, extracellular matrix (ECM) and cell cycle transcriptional programs were disrupted. Accordingly, in the hippocampi of adolescent Shank3∆11(−/−) mice we found proteins of the collagen family and core cell cycle proteins downregulated. In vitro Li treatment of Shank3∆11(−/−) cells had a rescue-like effect on the ECM and cell cycle gene sets. Reversed ECM gene sets were part of a network, regulated by common transcription factors (TF) such as cAMP responsive element binding protein 1 (CREB1) and β-Catenin (CTNNB1), which are known downstream effectors of synaptic activity and targets of Li. These TFs were less abundant and/or hypo-phosphorylated in hippocampi of Shank3∆11(−/−) mice and could be rescued with Li in vitro and in vivo. Our investigations suggest the ECM compartment and cell cycle genes as new players in the pathophysiology of Shank3 deficiency, and imply involvement of transcriptional regulators, which can be modulated by Li. This work supports Li as potential drug in the management of PMDS symptoms, where a Phase III study is ongoing.

## Introduction

ASD typically manifests during early childhood and causes persistent alterations in social interaction and communication, restricted repetitive behaviors, as well as sensory anomalies. The co-occurrence of ID, functional language impairment [1], and psychiatric disorders like depression is common [2]. Although ASD is considered a neurodevelopmental disorder with a multifactorial etiology including genetic and environmental factors, several ASD risk genes have been identified [3, 4] of which many encode synaptic proteins.

One gene frequently associated with ASD and ID is Shank3, mutated in more than 2 % of collective patients [5]. SHANK3 is known for its post-synaptic function as a major scaffolding protein for a variety of other synaptic proteins, including PSD95, GRM5, NMDA, AMPA and SHARPIN, as well as its enrichment in a range of brain regions like the cortical layer 2-4, the hippocampus and the striatum (reviewed by Monteiro and Feng [6] and Delling and Boeckers [7]). Interaction with the cytoskeleton has been proposed as a shared function with other ASD-associated proteins like ADNP [8]. Several SHANK3 mutant mouse models have been generated, and presented with heterogenic ASD-like phenotypes (reviewed by Delling and Boeckers [7]), arising in a brain area related manner [9]. In humans, Shank3 mutations commonly cause a syndromic form of ASD, the PMDS [10, 11]. Individuals with PMDS present with global developmental delay, delayed or absent speech, ID, ASD-like behaviors, neonatal muscular hypotonia, sensory anomalies and heterogenic facial dysmorphisms [11]. In early childhood or adolescence, these individuals can regress and lose previously acquired skills, along with the occurrence of psychiatric illnesses like catatonia, atypical bipolar disorders (BD) and other affective symptoms [12–17]. This is consistent with higher prevalence of BD and anxiety disorder in ASD [18], suggesting partially shared underlying mechanisms, but a genome-wide analysis of SNPs did not indicate a genetic correlation [19], and shared but differential involvement of ERK signaling has been discussed [20].

Advances have been made to understand the pathophysiology of Shank3-deficiency, identifying the putative role of Shank3 causative for the heterogenic symptoms. ASD-like symptoms and ID are linked to synaptic (dys-) function [21] (reviewed by Zoghbi and Bear [22]), with a well-established involvement of SHANK3 [23–25]. Also, functional brain connectivity anomalies and white matter alterations have been reported in ASD patients [26] as well as in Shank3-deficiency [27, 28]. This might be explained by delayed myelin maturation in the absent of SHANK3 and its expression in oligodendrocytes and Schwann cells [29]. Intriguingly, biological correlates have also been discovered for extra-cerebral symptoms of PMDS, like the neonatal muscular hypotonia, likely explained by impaired maturation of striated muscles and neuromuscular junctions (NMJ), with SHANK3 directly localized at Z-discs [30].

Gene expression regulation can be affected in ASD and a large portion of ASD-risk genes are involved in these processes [31, 32]. Likewise, SHANK3 has been associated with gene expression regulation, on the one hand by synaptic mechanisms like impaired CREB signaling [33–35] – though alterations in broad CREB target gene expression have not been shown – and on the other hand by dysregulation of general signaling pathways [36, 37]. Although, bulk transcriptome analysis in Shank3-deficiency models have been conducted across different species, tissues, time points and treatments [38–44], transcriptional patterns in the hippocampus have not been directly linked to regulatory elements nor have they been directly translated to the protein level.

The hippocampus contributes to deficits in memory, spatial reasoning, social interaction, and eventually repetitive behavior and restricted interests in ASD, and is also relevant in other psychiatric disorders like anxiety disorder or depression (reviewed by Banker et al. [45]). Coherently, mouse models of Shank3-deficincy exhibit impaired social learning, repetitive behavior and increased fear, and despite the pronounced localization of Shank3 to cortico-striatal synapses, hippocampal synapses are structurally and functionally altered (reviewed by Delling and Boeckers [7]).

There is no causal treatment for PMDS available at the moment, though pharmacological substances show beneficial effects on Shank3-defient phenotypes (reviewed by Delling and Boeckers [7]). Interestingly, lithium (Li) treatment of Shank3-deficient individuals leads to clinical improvement in single cases, especially stabilization of mood and behavior, as well as reduction of clinical regression [46, 47]. Li is the first line treatment for the prevention of depressive and manic/hypomanic episodes in bipolar disorder (BD) [48], as well as a viable mood stabilizer in ASD [49, 50]. Notably, serum levels of Li are decreased in children with ASD [51] and correlate with ASD severity [52]. Considering the promising case reports of Li as mood stabilizer in PMDS, and improving affective symptoms in ASD, a Phase III study has been started in 2022, treating SHANK3-haploinsufficient cohorts with Li (ClinicalTrials.gov identifier: NCT04623398). Since the exact mechanism of action of Li in improving affective symptoms in PMDS and ASD is not known, it is crucial to investigate the effect of Li on Shank3-deficiency, to explain clinical improvement and to develop further treatment options.

In this study we aim to (i) comprehensively explore the Shank3-deficient transcriptome in hippocampal cells to (ii) find biological functions impaired in Shank3-deficiency besides synaptic function and (iii) test the effects of Li on potential transcriptional alterations, as well as (iiii) identify underlying transcriptional regulators.

## Methods

### Animals

The Shank3Δ11−/− mice (C56BL/6 genetic background) breeding was performed as described previously [53]. Animals were kept at constant temperature (22 ± 1 °C) and humidity (50%) with a 12 h light/dark cycle and provided with food and water ad libitum. C56BL/6 mice were used as controls.

### Lithium treatment

A 100 mmol/L Li stock solution was prepared by adding 37 mg lithium (Li) carbonate to 10 ml filtered (20 μm) Millipore water. At DIV 9 medium was replaced by a mix of fresh Neurobasal+++ and Li stock solution to the final Li concentration. A treatment duration of 5 days was adapted, since it sufficed to rescue Shank3-deficient phenotypes in previous in vitro experiments [30]. The optimal concentration of Li for the long-term treatment of bipolar disorders ranges between 0.6 mmol/L and 0.75 mmol/L but can be higher for patients with mostly manic episodes [54]. Moreover, low doses of Li promise to exhibit various positive effects on the brain [55]. Since Li treatment is associated with serious adverse effects even in the therapeutic range [56], a low Li concentration of 0.1 mmol/L was used, to evaluate the effects in sensitive RNA sequencing experiments. To reliably confirm RNA sequencing results a higher Li concentration of 1 mmol/l was used for immunocytochemistry analysis.

For in vivo lithium treatment male Shank3Δ11(−/−) mice were single housed under standard laboratory conditions with temperatures at 22 ± 1 °C and 50% ±10% humidity and a 12 h dark/light cycle. All animal experiments were performed in compliance with the guidelines for the welfare of experimental animals issued by the Federal Government of Germany and approved by the local ethics committee at Ulm University and the Regierungspräsidium Tübingen with the ID numbers: 1595. Mice received either control food and regular drinking water or lithium-supplemented diet containing 0.3% (w/w) lithium carbonate ad libitum. To counteract potential toxicity of lithium, mice fed with the lithium diet received drinking water with 1.5 % (w/w) sodium chloride. Treatment started at postnatal day (P) 28 and mice were sacrificed at P60-P62 by cervical dislocation and brains were collected for biochemical analysis.

### RNA isolation and bulk RNA sequencing

See Supplementary Information for details.

### DEG analysis

All data analysis computations were performed using R version 4.1.0 and RStudio 2022.12.0 + 353. Mainly following [57], functions from the limma [58] and edgeR [59] package were used for DEG analysis. Only genes and corresponding read counts with entrezID (determined from Ensemble ID with the biomaRt R package) and RNA biotype of protein_coding, lincRNA, Mt_rRNA, snoRNA, miRNA, snRNA, scaRNA, rRNA, ribozyme or macro_lncRNA were included in the analysis. Read counts were normalized using edgeR::calcNormFactors() with “TMM” method. Genes were filtered with edgeR::filterByExpr() for genes with minimum 15 counts in 25 % of the samples. Batch effects were removed either for graphical representation with limma::removeBatchEffect() and visualized as MDS plots based on limma::plotMDS data or for differential gene expression analysis as factor in the linear model (LM). A voom object was created from the filtered genes and a design based on the following formula:

$$\sim 0+{{{{{\rm{group}}}}}}+{{{{{\rm{batch}}}}}}$$

where the group factor encodes WT_Veh, WT_Li, KO_Veh and KO_Li, and the batch factor encodes the 4 experimental batches. From this design the contrasts were derived using limma::makeContrasts(). Next, a LM was fit to each gene with limma::lmFit() and contrasts were computed from the LMs using limma::contrast.fit(). Finally, limma::eBayes() was used to calculate Empirical Bayes statistics based on the LMs. Differentially expressed genes were identified with Limma::decideTests() with Benjamini–Hochberg (BH) adjusted *p* values < 0.01 and |logFCs| > log2(1.2).

### Gene ontology

Gene ontology analysis on DEGs was performed using the clusterProfiler R package. All previously included genes were provided as background genes, and only terms with a least 10 genes were included, *p* values were BH-adjusted, and *q* values were calculated. Terms were considered enriched when *p* values and *q* values were <0.01. Parent terms were identified with the rrvgo package using “wang” method to calculate the semantic similarity and terms were weighted by -log10(q value) when reduced to parent terms. Jaccard similarities were calculated with stats::dist(). Community detection was performed with the tidygraph::group_louvain() function.

### EGSEA

Gene set enrichment was tested with the EGSEA R using the EGSEA::egsea() function. This method combines multiple gene set enrichment analysis methods to improve performance [60]. We included following methods: “ora”, “gage”, “camera”, “gsva”, “padog”, “safe”, “plage”, “zscore”, “ssgsea”, “globaltest”, and “fry”. The EGSEA::buildIdx() function was used to access annotated gene sets from the Gene Set Data Base Gene Ontology with a minimum of 10 genes. Gene sets from CC, MF, BP and the TFactS catalog [61] with adjusted *p* values < 0.05 were selected. The differences between the logFC of GS pairs from the genotype and treatment contrast were calculated:

$${\triangle \log {{{{{\rm{FC}}}}}}}_{{{{{{\rm{GS}}}}}}}={{{{{\rm{|}}}}}}\log {{{{{\rm{FC}}}}}}({{{{{{\rm{GS}}}}}}}_{{{{{{\rm{treatment}}}}}}})-\log {{{{{\rm{FC}}}}}}({{{{{{\rm{GS}}}}}}}_{{{{{{\rm{genotype}}}}}}}){{{{{\rm{|}}}}}}$$

From the median.rank values supplied by EGSEA::egsea(), the mean rank of each GS pair was calculated:

$${{{{{\rm{X}}}}}}({{{{{\rm{rank}}}}}})=({{{{{\rm{median}}}}}}.{{{{{\rm{rank}}}}}}({{{{{\rm{treatment}}}}}})+{{{{{\rm{median}}}}}}.{{{{{\rm{rank}}}}}}({{{{{\rm{genotype}}}}}}))/2$$

Jaccard similarities between GS pairs were calculated with stats::dist().

### Image acquisition

See Supplementary Information for details.

### Image analysis

See Supplementary Information for details.

### Immunocytochemistry (ICC)

See Supplementary Information for details.

### Immunohistochemistry (IHC)

See Supplementary Information for details.

### Plots and statistical analysis

All figures and plots were generated in R with ggplot2, pheatmap, ggVennDiagram, tidygraph and ggraph packages, as well as Adobe Illustrator CS6. For two group comparisons, data was tested for normal distribution using rstatix::shapiro_test(). If both groups passed, rstatix::t_test() was used to compare groups, otherwise rstatix::wilcox_test() was used. For comparisons of more than two groups, data was tested for homogeneity of variance using rstatix::levene_test(). If given, ANOVAs were computed with rstatix::anova_test() and post-hoc tests with rstatix::tukey_hsd(), otherwise rstatix::kruskal_test() was used. If compared samples were paired in the experimental process, pair-wise statistical tests were performed accordingly. If these pairs were acquired from several batches, z-transformation was used for data visualization while the statistical tests were performed on the otherwise normalized data.

### Primary and secondary antibodies list

See Supplementary Information (Supplementary Tables S1 and S2).

### Primary hippocampal cell culture

See Supplementary Information for details.

### Protein Isolation and Western Blot (WB)

See Supplementary Information for details.

### String

See Supplementary Information for details.

## Results

### Divergence of global gene expression in Shank3∆11(−/−)

**Fig. 1:**
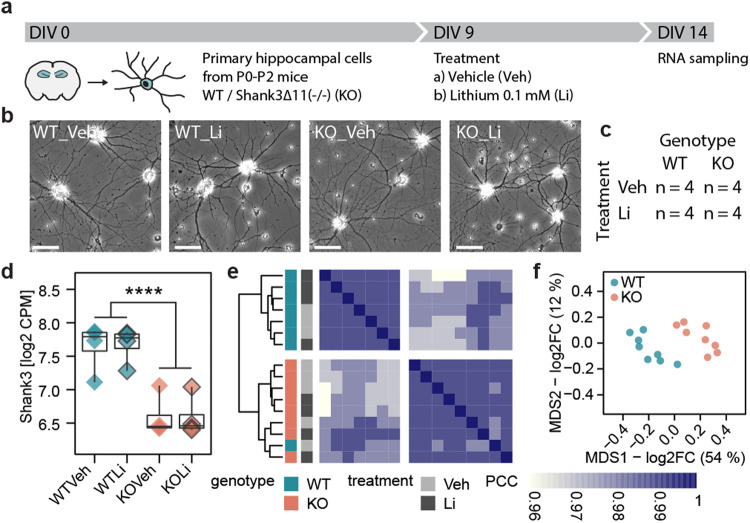
Divergence of global gene expression in Shank3∆11(−/−). **a** Experimental timeline. **b** Representative brightfield images of the cell cultures. Scale bar = 10 µm. **c** Cross table overview of groups and sample size. **d** Shank3 expression as log2 CPM. (genotype: *F*(1, 12) = 46.962, p**** < 0.0001; treatment: *F*(1, 12) = 0.006, *p* = 0.941; interaction: *F*(1, 12) = 0.013, *p* = 0.911; *n* = 4 per group, two-way ANOVA). **e** Hierarchical clustering of samples based on inter-sample PCCs. Samples labeled by genotype and treatment. **f** MDS plot of all samples, labeled by genotype. Axis correspond to leading log2FC. MDS1 accounts for 54 % of the similarity and MDS2 for 12%.

To explore the impact of Shank3 deficiency on the in-vitro transcriptome, we performed bulk RNA sequencing of cultured primary cells from hippocampi of wild type (WT) and Shank3∆11(−/−) (KO) P0-2 mice at day in vitro (DIV) 14 (*N* = 16), where DIV 14 represents a cell culture of stage 4/5 neurons with complex dendritic branches and synaptic connections [62]. At DIV 9 the cell cultures of each genotype were treated with Li or H2O (Vehicle/Veh) for 5 days (Fig. 1a, b). Consequently, four experimental groups were formed: WTVeh, WTLi, KOVeh, KOLi (each *n* = 4) (Fig. 1c).

Since experiments were carried out in four batches, we removed batch effects (Details in methods section) to effectively compensate batch-related clustering in Multi-Dimensional Scaling (MDS, see ref. [57] for details) (Supplementary Fig. S1a).

Shank3 transcripts were significantly less abundant in Shank3∆11(−/−) cells compared to WT, although in the Shank3∆11(−/−) model Shank3 loss is limited to Shank3a, Shank3b and Shank3c isoforms [63] (Supplementary Fig. S1b, modified from ref. [63]). Li treatment did not change Shank3 transcript abundance in both genotypes (Fig. 1d).

Pearson Correlation Coefficient (PCC) was above 0.95 between all samples, indicating high similarity in general gene expression, reflecting the samples’ shared hippocampal origin and genetic background. Although samples clustered by genotype in hierarchical clustering (Fig. 1e), and in MDS (Fig. 1f), they did not cluster by treatment group, suggesting a higher impact of Shank3 deficiency than Li treatment on overall gene expression. One WTVeh sample was identified as an outlier by hierarchical clustering of PCCs, however separated in MDS from the cloud of KO samples and was thus included in downstream analysis (Fig. 1e).

In conclusion, we confirmed reduced Shank3 transcript abundance in Shank3∆11(−/−) hippocampal cells and showed that broad gene expression differed between WT and Shank3∆11(−/−) but less between treatment groups.

### Disruption of extracellular matrix, cytoskeleton and cell cycle transcriptional programs in Shank3∆11(−/−)

**Fig. 2:**
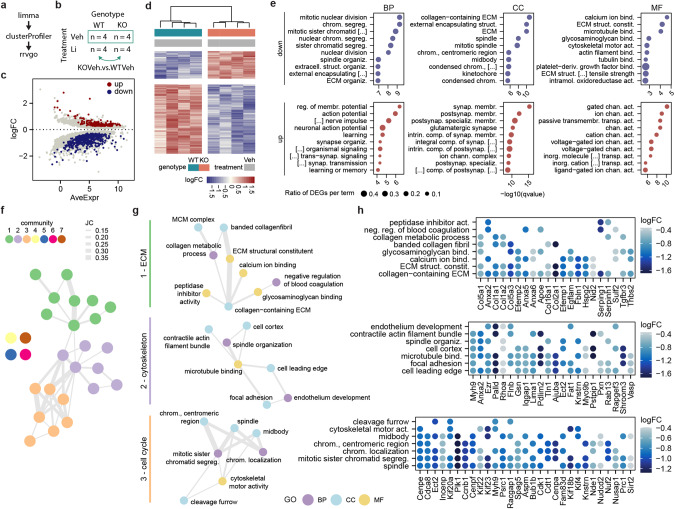
Disruption of extracellular matrix, cytoskeleton and cell cycle transcriptional programs in Shank3∆11(−/−). **a** R package pipeline. **b** Cross table, the groups of the analyzed contrast are labeled by the green box. **c** MA plot filtered for the top 5000 genes with the lowest adjusted *p* value (p.adj). **d** Hierarchical clustering of the contrasts’ samples based on logFCs of DEGs. **e** Top 10 enriched GO terms from up- and downregulated DEGs by -log10(qvalue). Size of points corresponds to the ratio of DEGs to all genes of the GO term. **f** Network of parent terms. Nodes represented parent terms and edges were drawn for JC > 0.1 and only between parent terms of different GOs. Colors correspond to communities detected by Louvain method. **g** Separated ECM (green bar), cytoskeleton (violet bar) and cell cycle (orange bar) communities. Node color corresponds to parent terms’ GO. **h** Key genes of the communities 1–3, defined as DEGs shared by three parent terms at least.

For a comprehensive analysis of the Shank3∆11(−/−) transcriptome from single differentially expressed genes (DEG) to overarching alterations in biological function we utilized a pipeline including limma for DEGs analysis, clusterProfiler for enrichment analysis of gene ontology (GO) terms and rrvgo to find semantic similarities between GO terms (Fig. 2a).

First, we performed DEGs analysis for the contrast KOVeh versus WTVeh (Fig. 2b). MA (log ratio – mean average) plot showed a fan-like distribution of genes along the Average Expression (AveExpr) axis as expected, but with a shift to more negative log2 fold changes (logFC). Accordingly, the number of downregulated genes (*n* = 492) exceeded those of upregulated genes (*n* = 195) (Fig. 2c). Based on logFC of these DEGs, hierarchical clustering clearly separated KOVeh and WTVeh samples, indicating consistent differential gene expression in the Shank3∆11(−/−) samples (Fig. 2d).

Next, we performed gene enrichment analysis of the GO terms cellular compartment (CC), molecular function (MF) and biological process (BP) on the down- and upregulated DEGs, respectively. From the downregulated DEGs we found 192 enriched GO terms with the top-ranking ones predominantly involved in mitosis, extracellular matrix (ECM), and cytoskeletal function. Highest ranking by *q*-value was mitotic nuclear division and chromosome segregation in BP as well as collagen-containing ECM and external encapsulating structure in CC. Three terms of MF had notably lower qvalues: calcium ion binding, ECM structural constituent and microtubule binding (Fig. 2e). However, the 114 enriched terms from the upregulated DEGs were in contrast mainly connected to synaptic transmission, synaptic membranes, and ion channels. In BP, regulation of membrane potential and action potential were the highest-ranking terms. CC terms with lowest qvalues were synaptic membrane and postsynaptic membrane. Gated channel activity and ion channel activity were the top terms of MF (Fig. 2e).

Here, we focused on the novel aspects of Shank3-deficency like dysregulation in cell cycle and ECM related gene expression, thus selecting the downregulated DEGs for further exploration. To investigate the biological functions disturbed in Shank3∆11(−/−) hippocampal cells, we used “rrvgo” to reduce redundance in the list of downregulated GO terms without manual selection. By incorporating Wang semantic similarities, enriched GO terms were grouped and mapped to common parent terms of the gene ontology trees. This approach identified 27 parent terms. These included cell cycle related parent terms like mitotic sister chromatid segregation, spindle organization and midbody as well as ECM related parent terms like ECM structural constituent and collagen-containing ECM, and cytoskeleton-related parent terms e.g., microtubule binding and cytoskeletal motor activity (Supplementary Fig. S2a).

So far, CC, MF and BP have been processed in parallel. To disentangle redundance between the GOs we constructed a network of parent terms based on the proportion of shared DEGs, as measured by Jaccard similarity coefficient (JC) (Supplementary Fig. S2b), only allowing edges between parent terms of different GOs surpassing a JC threshold. Using community detection, we identified three main communities and four single parent terms not connected to any other (Fig. 2f, fully labeled: Supplementary Fig. S2c). Community one essentially represented ECM related parent terms like collagen-containing, ECM structural constituent, collagen metabolic process, banded collagen fibril, glycosaminoglycan binding, peptidase inhibitor activity, calcium ion binding, negative regulation of blood coagulation and MCM complex. Community two comprised parent terms associated mainly with the cytoskeleton like microtubule binding, focal adhesion, cell leading edge, endothelial development, cell cortex, spindle organization and contractile actin filament bundle. Parent terms linked to the cell cycle were gathered in the third community, specifically mitotic sister chromatid segregation, chromosome centromeric region, chromosome localization, spindle, midbody, cleavage furrow and cytoskeletal motor activity (Fig. 2g). The cytoskeleton and cell cycle communities appeared strongly connected through the parent terms spindle organization and microtubule binding.

We defined the most frequently shared genes between parent terms, with a logFC below −0.4, as key genes of the community. In the ECM community these key genes belong overwhelmingly to the Collagen family (Col1/2/5/16) and the Annexin family (Anxa2/5/6) but as well included Efemp1/2, Apoe and Tgfbr3. Key genes of the cytoskeleton community were Myh9, Ezr, Palld and Rhoa among others. Both communities shared Anxa2 as key gene. Genes involved in cell cycle regulation like Plk1 and members of the Kif family (Kif4/20a/22/23/18b) were found to be key genes in the third, cell cycle, community (Fig. 2h).

To assess if these changes are specific for the Shank3∆11(−/−) mutation and the hippocampus and are Shank3 gene dosage dependent, we compared the DEGs with DEGs of published transcriptomic data sets from different brain regions of Shank3 KO mice lacking the Exons 14–16 (Yoo et al.) [41] and Shank3 overexpressing transgenic mice (Shank3TG) (Jin et al.) [64, 65] (Supplementary Fig. S2d–i). Shank3 was a DEG in all data sets. Only 1–5 DEGs overlapped with the Shank3 KO Exons 14–16 data sets (Supplementary Fig. S2g–i). The most DEGs were shared with the Shank3 TG data set of the medial prefrontal cortex (mPFC). Notably, in the Shank3 TG mPFC data set, the ECM associated DEGs Col1a1, Igfbp2 and Lama5 were overexpressed, in contrast to the Shank3∆11(−/−) data set (Supplementary Fig. S2d).

To summarize, here we were able to identify the disruption of three transcriptional programs in Shank3∆11(−/−) cells, representing three interconnected domains of cellular functions: The ECM, cytoskeleton, and cell cycle.

### In vivo alterations of ECM proteins in the hippocampi of Shank3∆11(−/−) mice

**Fig. 3:**
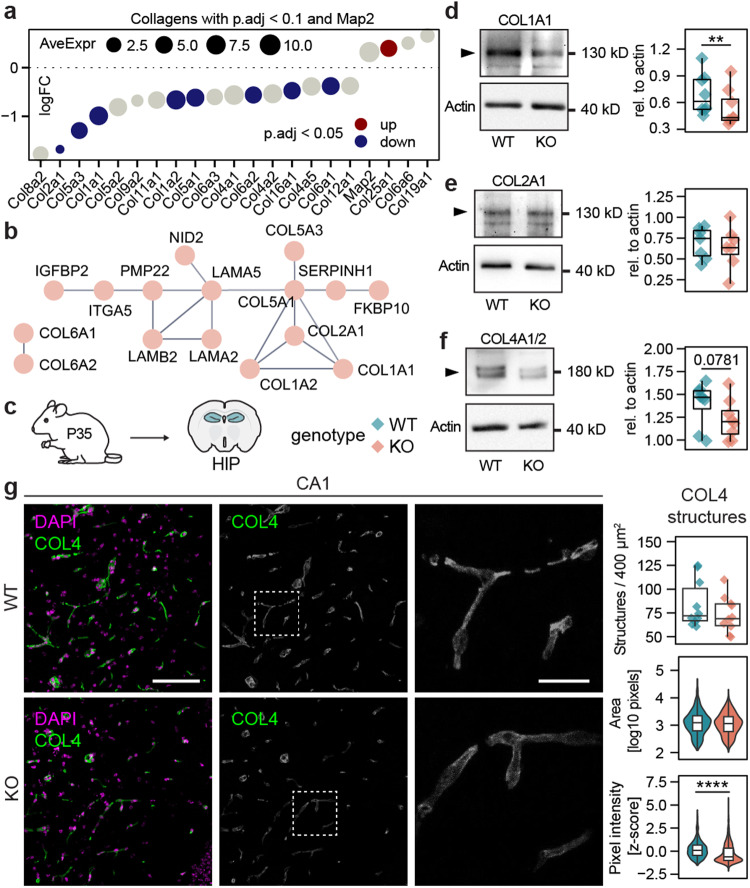
In vivo alterations of ECM proteins in the hippocampi of Shank3∆11(−/−) mice. **a** Collagens expressed in the cell cultures with p.adj < 0.1 and Map2. **b** Networks connecting collagens and other ECM related proteins, isolated from the STRING PPI network of all downregulated DEGs. **c** Pictogram of the tissue origin and genotype legend for **d**–**g**. Hippocampi of P35 Shank3∆11(−/−) and WT animals were used for protein isolation and subsequent WB analysis or coronary sectioned for IHC. **d** WB of COL1A1 and amounts relative (rel.) to actin (Z = 0, p** = 0.00781, *n* = 8 animals per genotype, paired wilcox rank test). **e** WB of COL2A1 and amounts rel. to actin (*t* = −1.015094369, df = 6, *p* = 0.349, *n* = 7 animals per genotype, paired two-sided t-test). **f** WB of COL4A1/2 and amounts rel. to actin (*Z* = 5, *p* = 0.0781, *n* = 8 animals per genotype, paired two-sided wilcox rank test). **g** Representative images from the CA1 hippocampal region stained for DAPI and COL4, and the analysis of the COL4 structures (number of structures: *Z* = 39, *p* = 0.427, *n* = 10 ROIs from 5 animals per genotype, paired two-sided wilcox rank test; area: *Z* = 294277, *p* = 0.131, *n* = 840(WT)/733(KO) structures from 5 animals per genotype, paired two-sided wilcox rank test; pixel intensity: *Z* = 209273, p**** < 0.0001, *n* = 840(WT)/733(KO) structures from 5 animals per genotype, paired two-sided wilcox rank test), scale bar = 100 µm, scale bar zoom = 25 µm.

Several genes from the collagen family were found to be expressed in Shank3∆11(−/−) and WT cell cultures, and most were expressed lower in comparison to Shank3∆11(−/−). While Col1a1, Col1a2, Col2a1, Col5a1, Col5a3, Col6a1, Col6a2 and Col16a1 were significantly under-expressed, Col25a1 was significantly overexpressed. For relation, across all samples AveExpr of Map2 was similar to most collagens, though Col2a1 had a notably lower AveExpr than Map2 and other collagens (Fig. 3a). To find predicted physical interaction partners of collagens proteins among all downregulated DEGs, we built a STRING Protein-Protein-Interaction (PPI) network from all downregulated DEGs and extracted one isolated community of 14 ECM-related proteins in addition to COL6A1 and COL6A2 forming another isolated cluster. COL1 and COL2a1 were predicted to connect to LAMA5, LAMA2 and LAMB2 via COL5a1. Interestingly, while IGFBP2-derived peptides were reported to rescue defects in a Shank3-deficent mouse model [66], IGFBP2 was part of the network too (Fig. 3b).

Since findings from cell cultures will not certainly translate into in vivo models and RNA transcript abundance does not necessarily reflect protein amounts, we examined the hippocampi from 35 postnatal days old (P35) WT and Shank3∆11(−/−) mice to confirm alterations in collagen expression (Fig. 3c). P35 mice are considered to reflect human adolescence [67] and at this age Shank3∆11(−/−) mice exhibit ASD associated stereotypical and repetitive behavior, as well as affective symptoms like avoidance and anxiety [68]. In addition Shank3∆11(−/−) mice present a deficient synaptic phenotype including altered synaptic protein composition [63] and ultra structural changes of the post synaptic density [53] Western blot (WB) analysis showed that in Shank3∆11(−/−) COL1a1 was significantly less abundant, and COL4a1/2 was reduced in comparison to WT. COL2a1 was unchanged (Fig. 3d–f). Likewise, the overexpressed transcripts of Col25a1 did not translate into increased protein amounts of COL25a1 (Supplementary Fig. S3a).

Vascular function and neuronal activity are closely connected, therefore alterations in COL4, a main component of the base membrane of blood vessels, is of particular interest. COL4 fluorescence staining of hippocampal slices of P35 mice resembled vessel-like structures, which showed reduced COL4 signal intensity in Shank3∆11(−/−), while the signal areas and number of structures did not differ between genotypes (Fig. 3g).

In conclusion, we found several collagens downregulated in Shank3∆11(−/−) hippocampal cell cultures as well as COL1 and COL4 reduced in the hippocampi of Shank3∆11(−/−) mice.

### In vivo alterations of cell cycle proteins in the hippocampi of Shank3∆11(−/−) mice

**Fig. 4:**
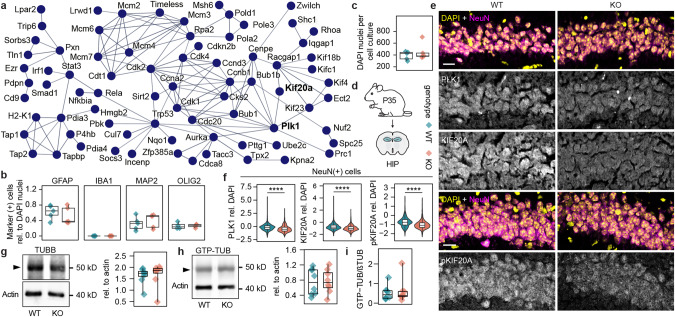
In vivo alterations of cell cycle proteins in the hippocampi of Shank3∆11(−/−) mice. **a** Network connecting cell cycle related proteins, isolated from the STRING PPI network of all downregulated DEGs. PLK1 and KIF20A are labeled bold. **b** Ratios of marker positive cells to DAPI nuclei (GFAP: *t* = −0.743700079, df = 7.687872835, *p* = 0.479, *n* = 5 cell cultures per genotype, two-tailed t-test; IBA1: *Z* = 13, *p* = 1, *n* = 5 cell cultures per genotype, two-tailed wilcox rank test; MAP2: *t* = 0.672397714, df = 7.973620187, *p* = 0.52, *n* = 5 cell cultures per genotype, two-tailed t-test; OLIG2: *t* = 0.028751815, df = 4.643111315, *p* = 0.978, *n* = 5 cell cultures per genotype, two-tailed t-test). **c** DAPI nuclei per cell culture (*Z* = 14, *p* = 0.841, *n* = 5 cell cultures per genotype, two-tailed wilcox rank test). **d** Pictogram of the tissue origin and genotype legend for **b**, **c**, **f**–**i**. Hippocampi of P35 Shank3∆11(−/−) and WT animals were used for protein isolation and subsequent WB analysis or coronary sectioned for IHC. **e** Representative images from the CA1 hippocampal region stained for DAPI, NeuN and PLK1/KIF20A/pKIF20A, scale bar = 20 µm. **f** PLK1, KIF20A and pKIF20A log2 of signal intensity rel. to DAPI signal in single cells (PLK1: Z = 448875, p**** < 0.0001, *n* = 1092(WT)/1196(KO) from 5 animals per genotype, two-tailed wilcox rank test; KIF20A: *Z* = 464471, p**** < 0.0001, *n* = 1092 (KO)/1196(KO) from 5 animals per genotype, two-tailed wilcox rank test); pKIF20A: *Z* = 557197, p**** < 0.0001, *n* = 1308(KO)/1342(KO) from 5 animals per genotype, two-tailed wilcox rank test. **g** WB of TUBB and amounts rel. to actin (*Z* = 20, *p* = 0.844, *n* = 8 animals per genotype, paired two-sided wilcox rank test). **h** WB of GTP-TUB and amounts rel. to actin (*t* = 0.47115966, df = 7, *p* = 0.652, *n* = 8 animals per genotype, paired two-sided t-test). **i** Ratios between GTP-TUB to TUBB values of the same animals. (*Z* = 0, *p* = 0.00781, *n* = 8 animals per genotype, paired two-sided wilcox rank test).

In the PPI network described above cell cycle associated genes formed a separate cluster including Plk1 and Kif20a (Fig. 4a), a kinase and kinesin involved in microtubule associated processes during cell cycle [69, 70]. Downregulated cell cycle genes suggest differences in cell numbers between genotypes, therefore we used common markers to label astrocytes (GFAP), microglia (IBA1), neurons (MAP2), oligodendrocytes (OLIG2), and all cell nuclei (DAPI) in the DIV14 cell cultures. As expected for post-mitotic neurons but surprisingly for the other cell types, the ratio between number of respective marker positive cells and all cell nuclei in the cultures did not depend on the genotype, indicating no effect of the downregulated genes on cell division rate after seeding at DIV0 (Fig. 4b). The total number of DAPI positive cells was not reduced in Shank3∆11(−/−) compared to WT either (Fig. 4c).

Again, we investigated the hippocampi of P35 mice from both genotypes to confirm results in vivo (Fig. 4d). We analyzed the PLK1, KIF20A and phospho-KIF20A (pKIF20A) fluorescence signals in the soma area of neurons relative to the cells’ DAPI signal in the CA1 region of hippocampal sections (Fig. 4e) and showed significant reduction in signal intensity for these proteins (Fig. 4f) translating the respective gene expression reduction into the in vivo protein amounts.

The dynamic instability of microtubes arises from a cycle of polymerization of GTP-bound beta-tubulin (TUBB) and alpha-tubulin monomers and the hydrolysation of TUBB-bound GTP to GDP. While GTP-TUBB monomers tend to polymerize, GDP-TUBB exhibit increased instability [71]. Inhibition of PLK1 can influence the dynamic instability of microtubules [72]. To assess microtubule turnover, we performed WB analysis of TUBB and GTP-TUB and found no difference in protein amounts (Fig. 4g, h) as well as GTP-TUB/TUBB ratio between genotypes (Fig. 4i), indicating unchanged tubulin dynamics.

These results suggest a downregulation of core cell cycle genes in Shank3∆11(−/−) with no effect on cell numbers and tubulin dynamics, therefore likely having no effect on cell division either.

### Li has rescue-like effect on disrupted transcriptional programs in Shank3∆11(−/−)

**Fig. 5:**
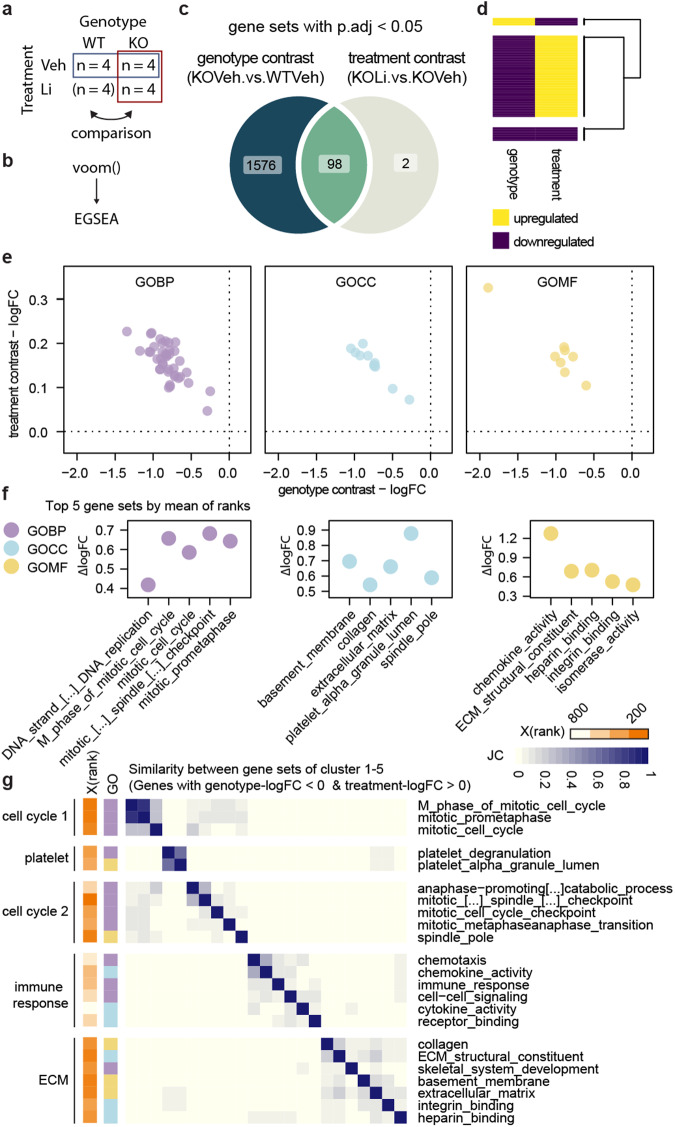
Lithium has rescue-like effect on disrupted transcriptional programs in Shank3∆11(−/−). **a** Cross table overview, the genotype contras (blue box), and the treatment contrast (red box) were compared. **b** R pipeline. **c** Venn diagram depicting the number of GSs enriched in both contrasts and uniquely in genotype or treatment contrast. **d** Hierarchical clustering of the overlapping GSs by the contrast based on the direction of regulation (up = 1, down = 0). **e** LogFC values of the overlapping GSs for the genotype and treatment contrast, separated by GO. **f** Difference of logFC between genotype and treatment contrast for the top 5 GSs by mean rank. Colors correspond to GO in **f** and **g**. **g** Clusters 1–5 of hierarchical clustering of shared GS based on inter-GS JC including only genes with logFC < 0 for genotype and >0 for treatment contrast. Orange gradient corresponds to mean rank of the GS.

Li is a potential drug candidate for the management of Shank3-deficiency [46, 47]. Here, we tested if the transcriptional programs disrupted in Shank3∆11(−/−) can be rescued by Li, by comparing the contrasts KOVeh versus WTVeh (genotype) and KOLi versus KOVeh (treatment) (Fig. 5a). No DEGs were detected for the treatment contrast (Supplementary Fig. S4a), therefore we utilized Ensemble of Gene Set Enrichment Analysis (EGSEA) [73] to perform gene set enrichment analysis (GSEA) on complete ranked gene lists for both contrasts. (Fig. 5b). Gene sets (GSs) from BP, MF, CC and Regulatory (REG) GOs were included. This resulted in 100 enriched GSs for the treatment contrast, of which 98 overlapped with 1674 enriched GSs of the genotype contrast (Fig. 5c). Most of the overlapping GSs (*n* = 78) were downregulated in the genotype contrast and upregulated in the treatment contrast, indicating a rescue-like effect of Li on these GSs (Fig. 5d).

Here, we selected those inversely regulated GSs to be further investigated. REG GSs were processed in parallel; the results are shown in Fig. 5a, b. Comparing the average logFC of this subset of GSs across contrasts and GOs revealed greater changes between genotypes than treatment groups, suggesting a partial reversal of the genotype-attributed downregulation by Li (Fig. 5e).

To find the most important GSs of the subset, we calculated the mean of both contrast ranks among all initially enriched GSs. Intriguingly, the top 5 GSs for BP, CC, and MF, respectively included mostly GSs related to cell cycle and ECM, matching the cellular functions we found to be predominantly affected by Shank3-deficency (Fig. 5f).

To find meaningful clusters among all GSs of the subset, we calculated the proportion of shared genes between GSs, including only genes inversely expressed between contrasts (negative logFC in genotype contrast, positive logFC in treatment contrast) and performed hierarchical clustering. This yielded 6 clusters of which clusters 1 to 5 were smaller, included GSs representing apparent domains of cellular function, ranked higher, and showed higher proportions of shared genes, in comparison to cluster 6 (Supplementary Fig. S4b). 12 of the 15 top ranking GSs from Fig. 3f were found in cluster 1 to 5. In line with our previous findings, two clusters represented cell cycle processes, the largest cluster comprised GSs related to ECM components and functions. In addition, Li inversed two GSs of platelet function, which was previously found to be relevant in the ECM parent term community of Shank3∆11(−/−) cell culture. Though not detected before, transcriptional programs involved in immune response were found to be diverting (Fig. 5g). Interestingly, no explicitly synaptic gene sets were rescued by Li (Supplementary Fig. S4b). Among the top 10 most shared genes were PLK1 in the cell cycle clusters and COL1A1/2 and COL4A3 in the ECM cluster (Supplementary Fig. S4c).

In summary we found that ECM and cell cycle related GS clusters were disrupted in Shank3∆11(−/−) cells and were partially rescued by Li.

### Common transcriptional regulators link Li-rescued clusters

**Fig. 6:**
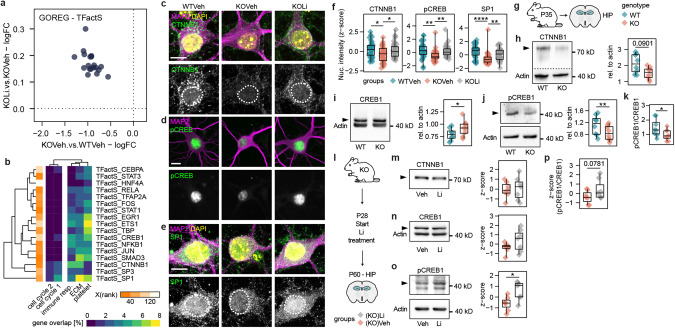
Common transcriptional regulators link Li-rescued clusters. **a** LogFC values of the inversed GSs from the TFacts catalog for the genotype and treatment contrast. **b** Hierarchical clustering of inversed TFactS GSs and the previously established clusters 1–5 of inverted GS from the GOs MF, CC, BP based on percentage of shared genes. Orange gradient corresponds to mean rank. **c** Representative images of primary hippocampal neurons stained for DAPI, MAP2 and CTNNB1, dotted lines = DAPI outlines, scale bar = 5 µm. **d** Representative images of primary hippocampal neurons stained for MAP2 and pCREB1, scale bar = 5 µm. **e** Representative images of primary hippocampal neurons stained for DAPI, MAP2 and SP1, dotted lines = DAPI outlines, scale bar = 5 µm. **f** Nuclear intensity of CTNNB1 and pCREB signals in primary hippocampal neurons. (CTNNB1: *F*(2,117) = 4.802, *p* = 0.01, *p = 0.0129 (WTVeh.vs.KOVeh), *p = 0.044 (KOVeh.vs.KOLi), *p* = 0.891 (WTVeh.vs.KOLi), *n* = 40 neurons per group from 4 independent experiments, one-way ANOVA followed by tukey-hsd post-hoc test; pCREB: *F*(2,108) = 4.802, *p* < 0.001, **p = 0.00181 (WTVeh.vs.KOVeh), **p = 0.00607 (KOVeh.vs.KOLi), *p* = 0.924 (WTVeh.vs.KOLi), *n* = 37 neurons per group from 4 independent experiments, one-way ANOVA followed by tukey-hsd post-hoc test); SP1: *F*(2,113) = 14.83, *p* < 0.001, ****p < 0.001 (WTVeh.vs.KOVeh), **p = 0.0098 (KOVeh.vs.KOLi), *p* = 0.0588 (WTVeh.vs.KOLi), *n* = 36–40 neurons per group from 4 independent experiments, one-way ANOVA followed by tukey-hsd post-hoc test). **g** Pictogram of the tissue origin and genotype legend for **h**–**k**. Hippocampi of P35 Shank3∆11(−/−) and WT animals were used for protein isolation and subsequent WB analysis. **h** WB of CTNNB1 and amounts rel. to actin (*t* = −1.965566362, df = 7, *p* = 0.0901, *n* = 8 animals per genotype, paired two-sided t-test). **i** WB of CREB1 and amounts rel. to actin (*t* = 2.496216754, df = 7, *p = 0.0412, *n* = 8 animals per genotype, paired two-sided t-test). **j** WB of pCREB1 and amounts rel. to actin (*t* = −3.928882749, df = 7, **p = 0.00568, *n* = 8 animals per genotype, paired two-sided t-test). **k** Ratios between pCREB to CREB1 values of the same animals (*t* = −3.393497057, df = 7, *p = 0.0115, *n* = 8 animals per genotype, paired two-sided t-test). **l** Pictogram of the experimental setup and legend for **m**–**p**. **m** WB of CTNNB1 and z-scored amounts rel. to actin (*t* = 0.042737218, df = 6, *p* = 0.967, *n* = 7 animals per group, two-sided paired t-test). **n** WB of CREB1 and z-scored amounts rel. to actin (*t* = 1.66076912292605, df = 6, *p* = 0.148, *n* = 7 animals per group, two-sided paired t-test). **o** WB of pCREB1 and z-scored amounts rel. to actin (*t* = 3.479384195, df = 6, *p = 0.0132, *n* = 7 animals per group, two-sided paired t-test). **p** Z-scored ratios between pCREB to CREB1 values of the same animals (Z = 25, df = 6, *p* = 0.0781, *n* = 7 animals per group, paired wilcox-test).

Next, we set out to find general targetable pathways connected to the rescued GS clusters. Therefore, we examined the REG GO GSs enriched in the previous step. These GSs contain genes whose regulation is attributed to certain regulatory elements like transcription factors (TFs) or miRNAs. Here we focused on TFs from the TFactS catalog. Comparing the average logFC of REG GSs between contrasts, made clear that they were downregulated to a greater extent between genotypes than upregulated between treatment groups (Fig. 6a) suggesting a partial rescue by Li as well. Intriguingly, β-Catenin (CTNNB1), that can be stabilized by Li [74], was found to be a TF GS downregulated between genotypes and upregulated between treatment groups, indicating a role for β-Catenin signaling in Shank3-deficiency and verifying Li action on the cell culture. Likewise, CREB1, JUN, NFKB1, SMAD3, SP1/2, and STAT1/3 were among the inverted REG GSs (Supplementary Fig. S5a).

Henceforth, we investigated REG GSs that overlap in gene content with the previously found inverted GS clusters from BP, CC and MF. Therefore, we collapsed each cluster to be one cluster GS containing all genes of its primary GSs and computed the Jaccard similarity among them and the REG GSs. Interestingly cell cycle cluster one and two did share almost no genes with almost all REG GSs besides minimal overlap with CTNNB1, SP1/2, and CREB1. ECM overlapped especially with SP1, SMAD3, and CTNNB1. Platelet cluster showed overlapping genes mainly with ETS1, SP1, EGR1, and TBP, while immune response overlapped the most with CTNNB1. Among the highest-ranking REG GS were CTNNB1, SMAD3, and STAT1/2 (Fig. 6b).

CTNNB1, CREB1 and SP1 involvement is of particular interest, considering their respective role in brain development [75], neuronal plasticity/memory [76] and neuronal degeneration [77]. To confirm their downregulation in Shank3∆11(−/−) cell culture and its rescue by Li, we performed immunocytochemistry analysis of CTNNB1, Ser133-phospho CREB (pCREB) and SP1 (Fig. 6c, d). In Shank3∆11(−/−) neurons treated with Veh CTNNB1 and pCREB signals were significantly decreased in comparison to WTVeh neurons, while Shank3∆11(−/−) neurons treated for 5 d with 1 mM Li exhibited increased CTNNB1, pCREB as well as SP1 signals in contrast to Shank3∆11(−/−) Veh controls (Fig. 6e).

To assess if the Li-rescued TFs translate into in-vivo models as possible targets for future treatment studies, we performed WB analysis of hippocampi from P35 Shank3∆11(−/−) and WT mice once again (Fig. 6f). Intriguingly, CTNNB1 amounts were reduced in Shank3∆11(−/−) (Fig. 6g). Total CREB amounts were significantly increased (Fig. 6h), while pCREB amounts were significantly decreased (Fig. 6i), resulting in a significantly lower pCREB/CREB ratio (Fig. 6j).

Next, we treated Shank3∆11(−/−) mice with Li and compared them to Shank3∆11(−/−) Veh controls, to evaluate the effect of Li on CTNNB1 and CREB1 in vivo. The Li treatment was started at P28 and at P60-62 the hippocampi were collected for WB analysis of CTNNB1 (Fig. 6l). The protein amounts of CTNNB1 were unchanged between Li and veh control group (Fig. 6m). Intriguingly, pCREB1 amounts were significantly increased, while CREB1 amounts were moderately increased in hippocampi of Li treated Shank3∆11(−/−) mice in comparison to the veh controls (Fig. 6n, o), resulting in an elevated pCREB1 to CREB1 ratio (Fig. 6p).

In brief, we showed that genes of the Li-rescued ECM cluster GS were connected to CTNNB1, CREB while genes of the cell cycle cluster GSs were rarely represented in the TF GSs. CTNNB1 and CREB were confirmed as targetable by Li, in Shank3∆11(−/−) cells and altered in Shank3∆11(−/−) hippocampi. In vivo Li treatment of Shank3∆11(−/−) mice rescued the phosphorylation of CREB1.

## Discussion

Here, we present an in-depth analysis of a Shank3-deficient transcriptome in a flexible and easy to treat in-vitro cell culture system, paired with evidence from adolescent Shank3-deficent mice, emphasizing novel aspects of Shank3-deficiency, namely disrupted ECM and cell cycle associated gene and protein expression. These changes were partially rescued by Li and ECM-associated genes appeared to be regulated by CTNNB1 and CREB1, which proved to be altered in vivo.

The ECM is scarcely researched in Shank3-defiency. In this study we found ECM alterations at a transcriptional level in cell cultures and protein level in adolescent mice, uncovering a reduction of collagen proteins, especially COL1 and COL4, expanding the previously limited indications of ECM involvement in Shank3-deficiency [38, 42, 78]. ECM proteins are produced in the CNS [79] and located to the vascular basement membranes and the interstitial matrix of the brain. They are involved in plasticity and myelination [80, 81]. Notably, Col1 induces cortex folding [82] and collagens may prevent immune cell invasion [83]. At the pre-synapse, Col4 is involved in synapse formation, maturation, and maintenance [84].

Comparing our results to previously published data, transcriptional signatures of ECM components and associated WNT (subsequent CTNNB1) signaling pathway are downregulated in human-induced pluripotent stem cell (hiPSC) -derived neurons from PMDS patients, though collagen under-expression was not reported [38]. In a transcriptome analysis of prefrontal cortex (PFC) of a Shank2 knock-out mouse at the age of 12 weeks, a collagen containing extracellular matrix term was reported to be downregulated as well [78]. In contrast, Col1 and Itga5 (which we both found to be under-expressed DEGs) have been described as over-expressed in an adult rat model of autism [85]. Additionally, an ECM term was found upregulated in adult Shank3B knock-out mice [42]. Summarized, evidence suggests a possibly age-dependent role for ECM components in the pathophysiology of Shank3-deficency, that needs to be addressed in further research. Comparing our Shank3∆11(−/−) transcriptomic data set with other published Shank3-deficient and Shank3 overexpressing data sets from different brain regions, suggested a hippocampus and Shank3∆11(−/−) specific effect. Intriguingly, the ECM related proteins Col1a1, Igfpb2 and Lama5 appear to be regulated in a Shank3 gene dosage dependent manner, indicating a broad relevance of ECM gene expression alterations in Shank3-deficiency.

Our Shank3∆11(−/−) mouse model lacks the ankyrin repeat region (ARR) of Shank3 (Supplementary Fig. S1b) [53]. This region contains a Ras interaction site, upon which mutations reduce the Rap1 and R-Ras bioavailability, in turn over-activating integrins [86]. This might connect Shank3-deficiency to ECM alterations, since integrins interact with collagens [87] and are involved in their assembly [88–90].

We report alterations of COL1 expression in Shank3∆11(−/−) mice which is remarkable, since syndromic forms of autism regularly co-occur and overlap in symptoms with connective tissue diseases from the Hypermobility Spectrum (HSD) and Ehlers-Danlos Syndromes (EDS) which involve mutations in collagens and other ECM-related genes [91]. Intriguingly, hyperextensibility was also reported in PMDS patients [92], suggesting ECM interruption as a shared feature of cognitive and connective tissue symptoms.

Part of the downregulated PPI network of ECM proteins, was peripheral myelin protein 22 (Pmp22) (Fig. 3b), a gene involved in formation and maintenance of myelin sheath in the nervous system [93–95]. Variation in gene dosage of Pmp22 can lead to Charcot-Marie-Tooth disease type 1A (CMT1A) [96] or Hereditary Neuropathy with Liability to Pressure Palsy (HNPP) [97], two inherited motor/sensory neuropathies. Likewise, PMDS patients can present with deficits in pain sensing [11]. Since myelination is altered in autism spectrum disease (ASD) (reviewed by Galvez-Contreras et al. [98]) and PMDS [29, 27], Pmp22 and connected ECM proteins potentially play a role in the pathophysiology. Altogether, this leads us to conclude that ECM, especially collagens, genes most likely contributes to common symptoms in PMDS, while the exact molecular processes need further investigation.

Regarding the second main finding of this study, the alterations in cell cycle genes, the in vitro cell culture results should be interpreted with care, since our cell cultures are a mixture of post-mitotic neurons and non-neuronal cell types. However, the in vivo analysis confirmed the alterations in post-mitotic neurons. Some of the core cell cycle genes we found downregulated in Shank3∆11(−/−) mice, are suspected to perform cell cycle non-related functions in post-mitotic neurons (reviewed by Frank and Tsai [99]) relating them to synaptic function in neurons. KIF20A executes functions relevant to mitotic brain cells in regulating cell cycle exit in neuronal progenitor cells [100] and post-mitotic brain cells like mediating IGF2BP3-bound mRNA transport along microtubules [101]. Also, PLK2 – a closely related kinase to PLK1 – is involved in homeostatic plasticity, relevant to post-mitotic neurons [102]. Remarkably, homeostatic plasticity is impaired in Shank3 KO mice and can be rescued by Li administration [102].

In the present study, we used Li to rescue ECM and cell-cycle related gene expression. Intriguingly, these where among the highest ranking disrupted transcriptional programs in Shank3∆11(−/−) cells. Previously, Li was reported to influence ECM processes, including collagen degradation [103] and immune cell extravasation [104]. As noted, the disruption of ECM-related gene expression may contribute to myelination deficits in Shank3-deficency. Interestingly, pharmacodynamic mechanisms of Li and other antipsychotics converge on signaling pathways affecting myelination and most recently ECM component stabilization preservation was linked to Li response [105].

Although, Li increased SHANK3 amounts in Shank3-haploinsufficient human embryonic stem cells (hESC) -derived neurons [106] and in human induced pluripotent stem cells (hiPSC) -derived motoneurons and myotubes, it had no effect on Shank3 transcript abundance in Shank3∆11(−/−) and WT hippocampal cells in our study, implying a post-transcriptional effect on SHANK3 amounts. Li treatment restored maturation of neuromuscular junction in co-cultures of hiPSC-derived motoneurons and human biopsy-derived myotubes [30] as well as rescuing overgrooming in Shank3 KO mice [102], indicating a benefit for Shank3-deficient phenotypes.

Here we add critical information on clusters of genes rescued by Li in Shank3∆11(−/−), what might be exploited for advanced treatments. We identified several TFs, involved in the regulation of Li-rescued genes, which in part proved to be altered in Shank3∆11(−/−) hippocampus in vivo. ECM-related genes overlapped particularly with genes regulated by CTNNB1, while CTNNB1 was reduced in Shank3∆11(−/−) hippocampi.

CTNNB1 pathway controls ECM constituents, regulates BBB maturation [107] and is involved in ASD [21, 108, 109]. Since, CTNNB1 switches on target gene expression when its degradation by phosphorylated GSK3 is inhibited and it is translocated to the nucleus [110], low CTNNB1 amounts explain reduced expression of CTNNB1-related genes. Reduced Akt signaling in SHANK1/3 double KO mice [37] and decreased Akt-dependent phosphorylation of GSK3 in Shank3 KO mice [36] may explain decreased CTNNB1 concentration in Shank3∆11(−/−). In addition, SHANK3 CTNNB1 interaction has been confirmed in qNSC, although there, Shank3 loss led to nuclear translocation of CTNNB1 in qNSC [111]. In addition, loss of Shank3 interaction with CTNNB1 via the PDZ domain, in a truncating mutation of Shank3, has been shown to negatively affect the downstream pathway [112]. We rescued CTNNB1-related gene expression with Li, which inhibits GSK3 [113], subsequently increasing CTNNB1 concentration [74]. Decreased CTNNB1 amounts in Shank3∆11(−/−) can potentially be explained by the loss of SHANK3 ankyrin repeat domains interacting with SHARPIN [114], which in turn would stabilize CTNNB1 [115] and additionally activate the Wnt/CTNNB1 pathway [116].

The CREB transcription factor is foremost known for its involvement in memory acquisition and its activation through neuronal activity (reviewed by Mayford and Kandel [76]). CREB1 activation through phosphorylation can have various region-specific effects, including modulation of depression, addiction, and anxiety (reviewed by Carlezon et al. [117]) and can be regulated by ERK signaling which is likely involved in autism and bipolar disorder (reviewed by Kalkman [20]). Outside the brain, CREB1 has been shown to regulate Col1 expression in rat hepatic stellate cells [118] and, in a complex with SMAD2/3, Col1 and Col3 expression in cardiac fibroblasts [119]. In Shank3 shRNA transfected hippocampal neurons, impaired GRM5 signaling leads to reduced phosphorylation of ERK1/2 and CREB1 [34]. In addition, a SHANK3-CaMKII interaction via Shank3 amino acid 829–1130 sustains CREB1 phosphorylation in cultured neurons [33, 35]. Since phosphorylation of CREB alone is not sufficient to predict the expression of target genes [120], we expand previous findings, by uncovering the downregulation of CREB target genes in Shank3∆11(−/−) in vitro, identifying them as ECM-related. We report hypo-phosphorylation of CREB1 in P35 Shank3∆11(−/−) mice, in contrast to hyperphosphorylation reported in 4-months-old SHANK3-KO mice [121], suggesting age-dependent alterations in CREB1 phosphorylation. Moreover, we established Li as viable option to partially rescue CREB target gene expression in Shank3∆11(−/−) neurons, in line with the literature proposing Li as activator of CREB1 (reviewed by Alda et al. [122, 123]). Confirmatory, in vivo Li treatment increased the phosphorylation of CREB1 in Shank3∆11(−/−) mice.

These results suggest that Li can rescue disrupted gene expression in the Shank3∆11(−/−) model through its well-established action on the GSK3-CTNNB1 signaling pathway [113], as well as Li dependent activation of CREB1 [123].

In contrast, SP1 has not been associated with gene expression dysregulation in Shank3-deficincy before. We report the downregulation of SP1 regulated genes in Shank3∆11(−/−) cells, which functionally annotated to ECM and cell cycle processes. SP1 signaling has been described as dysfunctional in post-mortem brains from ASD [124, 125] individuals and its transcription factor activity is essential to prevent degeneration of neurons by regulating microtubule related gene expression [77].

How Shank3 deficiency causes changes in gene expression remains to be fully unveiled, though our data, in the light of the most recent literature, supports some plausible mechanisms: By direct modulation of synaptic transmission and subsequent kinase dependent CREB activation and/or direct CREB activation, as well as CTNNB-SHANK3 interaction. Remarkably, no explicitly synaptic GS were rescued by Li, proposing a functional effect on synaptic transmission, and downstream elements like CREB and CTNNB1, rather than a direct effect on the synaptic protein composition. To expand on our results, multi-omics analysis of the Shank3∆11(−/−) genotype are needed, although we confirmed key findings of the bulk RNA sequencing on the protein level. RNA amounts might not correlate with respective protein amounts and phosphorylation and other post-translational modifications influence functions of genes. For simplification and straight forward discovery of new features of Shank3 deficiency we neglected synaptic genes in parts of our analysis, although a complex interplay between synaptic compartments and the ECM, as implicated by downregulated cadherins in Shank3 heterozygote brain organoids [126], is reasonable and needs detailed investigation. In this study we aimed to find overlooked molecular aspects of Shank3 deficiency and reported the disruption of ECM and cell cycle related gene expression, as well as their regulatory elements which can be rescued with Li. Each of these aspects require extended in-depth research.

### Supplementary information

Supplementary Material

Supplementary Material RNA Seq

## Data Availability

The RNA sequencing data was uploaded to the NCBI GEO database under the GEO accession number GSE248859.
