# Supplementary material for: Disrupted extracellular matrix and cell cycle genes in autism-associated Shank3 deficiency are targeted by lithium
Source: Mol Psychiatry. 2023 Dec 20;29(3):704–17. doi: 10.1038/s41380-023-02362-y (PMC11153165; doi:10.1038/s41380-023-02362-y)
Supplement: Supplementary file 1 — Supplementary Material [file 41380_2023_2362_MOESM1_ESM.docx]

Supplementary Information for

**Disrupted extracellular matrix and cell cycle genes in autism-associated Shank3 deficiency can be rescued with Lithium**

**Authors:** Valentin Ioannidis^1,#^, Rakshita Pandey^1,2,#^, Helen Friedericke Bauer^1,2^, Dr. Michael Schön^1^, PD Dr. Jürgen Bockmann^1^, Prof. Dr. Tobias M. Boeckers^1,3^, Dr. Anne-Kathrin Lutz^1,*^

**Affiliations:**

^1^ Institute for Anatomy and Cell Biology, Ulm University, 89081 Ulm, Germany

^2^ International Graduate School in Molecular Medicine Ulm, Ulm University, Ulm, Germany

^3^ German Center for Neurodegenerative Diseases (DZNE), Ulm site, 89081 Ulm, Germany

^# ­^Contributed equally

^*^ Corresponding author

**Correspondence:**

Dr. Anne-Kathrin Lutz
Ulm University, Institute for Anatomy and Cell Biology
Albert-Einstein-Allee 11, 89081 Ulm
Tel. 0049 731 50 23214

**Table of Contents**

Supplementary Materials and Methods……………………………………………………………..3

References……………………………………………………………………………………………..7

Supplementary Figures S1-4…………………………………………………………………………7

Supplementary Tables S1-S2………………………………………………………………………10

**Supplementary Materials and Methods**

**Primary hippocampal cell culture**

Ether 6-well plates (for RNA sequencing) or Glass coverslips placed into 24-well plates (for immunofluorescence staining) were coated with Gelatine 0,2 % for at least 2h at 37 °C. The Gelatine was removed and after 3 x 1 min washing-steps replaced with PLL for at least 6h at 37 °C. sterile water, plates were pre-equilibrated with DMEM+++ (20 % Ham’s Nutrient Mixture F12, 10 % FBS, 0,5 % Penicillin/Streptomycin, 0,5% Glutamine in DMEM) at 37 °C and 5 % CO2 until usage. P0-P2 SHANK3∆11(−/−) or WT mice, respectively, were decapitated, then the brains were removed carefully from the skull and placed in ice-cold HBSS. Under microscopic guidance, meninges were removed, and the hemispheres separated from the brainstem. 10-20 hippocampi were dissected and placed in ice-cold Hibernate-E medium supplemented with 2 % B27. Medium was removed and hippocampi were incubated with 2.5 % Trypsin in HBSS for 5-7 min at 37 °C followed by 4 x 1 min washing-steps with HBSS. HBSS was replaced with 5 % DNAse in HBSS. Then hippocampi were dissociated mechanically with a Pasteur pipette into single cells, filtered (100 μm), counted and resuspended DMEM+++. 30.000 cells/well were plated into 24-well plates and 300.000 cells/well into each 6-well plates. Medium was replaced after 3h with Neuobasal+++ (Neurobasal M supplemented with 2 % B27, 0.5 % Glutamine and 0.5 % Penicillin/Streptomycin). Medium was changed at DIV9 with Lithium treatment.

**RNA isolation and bulk RNA sequencing**

Qiagen RNeasy Mini kit was used to isolate total RNA according to the manufacturer’s description including all purification steps. RNA was eluted in 40 μl RNAse-free water. Quality control (QC), library construction, sequencing, and read mapping was performed by Novogen UK. In brief, messenger RNA was purified from total RNA and library cDNA synthesis was performed. Libraries were quality checked with Qubit and pooled for sequencing on Illumina platform. Raw data (raw reads) of fastq format were cleaned by removing reads containing adapter, ploy-N and low-quality reads. Reference genome index was built and paired-end clean reads were aligned to the reference genome using Hisat2 v2.0.5. Read counts were calculated with featureCounts v1.5.0-p3.

**Protein Isolation and Western Blot (WB)**

Hippocampal tissue from P35 male mice was extracted and homogenized in a buffer containing 0.32 M sucrose, 5 mM HEPES at pH = 7 with proteinase and phosphatase inhibitors. Equal amounts (7.5 µg) of protein were loaded in 8-10 % gels in pairs of WT and KO samples and separated at 140V for 1 h. The proteins were transferred onto a nitro-cellulose membrane, blocked with 5 % BSA in 0.1 % TBST or 5 % milk in 0.1 % TBST for 1 h at room temperature (RT). The membranes were incubated with the primary antibodies overnight at 4 ºC and incubated with their respective HR-coupled secondary antibodies for 1 hour at room temperature after washing 3x for 10 minutes with 0.2 % TBST solution. Membranes were imaged with Bio Rad Chemi Doc™ MP machine. Analysis was performed using ImageJ software version 1.52p and GelAnalyzer software version 2010a.

**Immunohistochemistry (IHC)**

12 µm thick cryosections of unfixed, frozen brains were collected on slides and fixed with 4 % PFA in 0.1 M sucrose solution for 15 min at RT. After 3x 5 min washing with PBS+/+, heat mediated antigen retrieval was performed using 0.1 M citric acid for 20 min at 95 ºC. The sections were blocked with 3% BSA and 0.2% Triton in PBS+/+ for 4 h at RT. The blocking solution was removed, and the membranes incubated with the primary antibody solutions for 48 hours at 4 ºC. After 3x 5 min washing with PBS +/+, respective secondary antibody solutions were added and incubated for 2 h at RT, then removed. DAPI 1:50,000 in PBS+/+ was added on the sections and incubated for 15 min at RT. After 3x 5 min washing with PBS +/+, the sections were mounted using VectaMount.

**Immunocytochemistry (ICC)**

Cultured cells were fixed at DIV14 with 4 % paraformaldehyde and 4% sucrose in 1x DPBS for 15 min at 37 °C, followed by 3 x 1 min washing-steps with DPBS. Permeabilization was performed with 0,2 % Triton X-100 for 10 min at RT. Subsequent, cultures were blocked with 10% goat serum or 10 % donkey serum and 5 % FBS in 1x DPBS for at least 3h at RT and incubated with primary antibodies in blocking buffer for 24h at 4 °C. After 4x washing-steps (1 min, 5 min, 15 min and 20 min) with 1x DPBS cultures were incubated with AlexaFlour® or DyLight®-coupled secondary antibodies in blocking buffer for 1h at RT and protected from light. Further washing steps (1 min, 10 min and at least 20 min) with 1x DPBS were performed before cultures were mounted with ProLong® Gold antifade reagent with or without DAPI.

**Image acquisition**

Brightfield images were acquired using the PAULA imaging system (Leica) with manual adjustment of brightness where needed. Conventional fluorescence images acquired with a upright Axioscope 2 microscope equipped with an Axiocam 506 mono camera and a Plan-Neofluar 20x or 40x oil immersion objective using the ZEN Blue software (Zeiss).

Confocal fluorescence images of neurons were acquired with a laser-scanning microscope (Leica DMi8) equipped with an ACS APO 40x, or 63× oil DIC immersion objective using the LasX software (Leica). Single images were captured with a zoom factor of 1.5, at a x-y resolution of 1,024 × 1,024 pixels, and a 0.346 μm step size along the z-axis covering the whole cell soma of cultured neurons or 2 μm of hippocampal slices. Tile scans were captured the same way with an ACS APO 20x oil DIC immersion objective covering the whole hippocampus.

**Image analysis**

ImageJ 1.52p was used for z-projection, ROI extraction, manual counting of cells, manual selection of nuclei, and measuring the mean intensity and size of areas.

To determine the number of cell types in the hippocampal cell cultures, we analyzed 10 images with the 20x objective per coverslip from two coverslips of each genotype in N = 4-5 independent cell cultures. We counted the marker positive cells manually, calculated the ratio to DAPI positive nuclei and plotted the mean for each cell culture. To compare total number of cells we summed the number of DAPI nuclei of all images per genotype and cell culture.

For the analysis of COL4 structures 400 μm x 400 μm regions of interest (ROIS) were selected directly beneath the CA1 cell band of the hippocampi, of both hemispheres’ tile scans of each animal. The EBImage R package [1] was used to segregate the structures and measure their size and pixel brightness.

To measure the PLK1 and KIF20A fluorescence signals in single neurons from the hippocampal CA1 region we analysed one image of the CA1 cell layer from each hemisphere and animal. EBImage R package functions were used to detect nuclei in the DAPI channels with adaptive thresholds and watershed. Then cell soma boundaries were detected in the NeuN channels using voronoi-segregation with the nuclei as seeds and the NeuN, and PLK1, respective KIF20A, mean pixel intensity was determined for each cell. Based on the log2 transformed areas and pixel intensities of NeuN, cells were categorized as NeuN positive or negative cells using k-means clustering. The log2-transformed ratio of PLK1, KIF20A and pKIF20A to DAPI was calculated.

We imaged 5 neurons (detected by MAP2 staining) per coverslip from two coverslips of each group in N = 3-4 independent experiments, to measure the nuclear TF signals in hippocampal cell cultures. The nuclei areas were selected manually ether in the DAPI staining or for pCREB directly in the pCREB channel. Mean pixel brightness of the nuclear areas were measured and z-transformed across all groups for each independent experiment.

**STRING**

The STRING web application was used to construct protein-protein interaction (PPI) networks with network type set to physical network, edges meaning confidence, text mining, experiments and databases as active interaction sources, minimum required interaction score of high confidence (0.7) and only queried proteins shown.

**Reference**

1. Pau G, Fuchs F, Sklyar O, Boutros M, Huber W. EBImage--an R package for image processing with applications to cellular phenotypes. *Bioinformatics* 2010; **26**(7)**:** 979-981.

**Supplementary Figures S1-5**

**Supplementary Figure S1. Batch effect removal and deleted isoforms in the Shank3∆11(−/−) mice**

a) MDS plot of all samples before and after removal of the batch effect, colors correspond to experimental batch and shape to genotype. Axis sow the leading log2FC. The percentage of similarity the MDS axis accounts for is shown in brackets. b) Illustration of the Shank3 gene, its exons, promotors, isoforms, and their molecular weight. The deleted SHANK3 isoforms in Shank3∆11(−/−) are labeled in grey.

**Supplementary Figure S2. Term assignment to parent terms based on Wang sematic similarity, hierarchical clustering, and community detection.**

mitotic_cell_cycle_checkpoint

mitotic_cell_cycle_spindle_assem

b

ly_checkpoint

mitotic_metaphaseanaphase_t

r

ansition

M_phase_of_mitotic_cell_cycle

mitotic_prometaphase

anaphase?promoting_[...]_protein_catabolic_process

spindle_pole

mitotic_cell_cycle

cytokine_activity

receptor_binding

chemotaxis

im

m

une_response

cell?cell_signaling

chemokine_activity

s

k

eletal_system_d

e

v

elopment

collagen

basement_memb

r

ane

e

xt

r

acellular_mat

r

ix

e

xt

r

acellular_mat

r

ix_st

r

uctu

r

al_constituent

platelet_deg

r

a

n

ulation

platelet_alpha_g

r

a

n

ule_lumen

hepa

r

in_binding

integ

r

in_binding

T

F

actS_CEB

P

A

T

F

actS_HNF4A

T

F

actS_S

TA

T3

T

F

actS_SP3

T

F

actS_T

F

AP2A

T

F

actS_CTNNB1

T

F

actS_CREB1

T

F

actS_ETS1

T

F

actS_FOS

T

F

actS_S

TA

T1

T

F

actS_JUN

T

F

actS_NFKB1

T

F

actS_RELA

T

F

actS_EGR1

T

F

actS_TBP

T

F

actS_SP1

T

F

actS_SMAD3

0

2

4

6

8

10

12

0.6

0.8

1

1.2

logFC

GOREG

KO

V

eh.v

s

.WT

V

eh

K

OLi.v

s

.

KO

V

eh

JUN

TBP

CEB

P

A

T

F

AP2A

EGR1

HNF4A

ETS1

CTNNB1

CREB1

SMAD3

SP1

S

TA

T3

S

TA

T1

FOS

SP3

NFKB1

RELA

**b**

**a**

a) Heatmaps of the Wang semantic similarity between the downregulated GO terms separated by GOs, their hierarchical clustering and assignment to parent terms. b) Hierarchical clustering of the parent terms based on their gene overlap measured as JC. c) Fully labeled network of parent terms from Fig. 2f. Nodes represent parent terms; edges represent JC between parent terms of different GOs; Colors represent communities detected with the Louvain method. d-i) Venn diagrams of shared DEGs between the Shank3∆11(−/−) transcriptomic data set and the Jin et. al 2018 and Yoo et. al 2022 data sets, separated by brain.region. logFC of the shared DEGs. CTX = Cortex, HIP = Hippocampus, mPFC = Medial prefrontal cortex, STR = Striatum.

**Supplementary Figure S3. Western Blot anaysis of COL25α1**

a) WB of COL25α1 amounts rel. to actin (t = 0.121545864, df = 7, p = 0.907, n = 8 animals per genotype, paired two-sided t-test).

**Supplementary Figure S4. Treatment contrast DEG analysis and** **hierarchical clustering of inversed GS from EGSEA based on JC.**

a) MA plot filtered for the top 5000 genes with the lowest adjusted p value (p.adj) for treatment contrast analysis. b) Hierarchical clustering of all inversed GS from the EGSEA analysis based on inter-GS JC. c) Top 10 mgenes with logFC < 0 in the gentoype contrast and logCF > 0 in the treatment contrast, most often shared by inversed GS in each cluster, color gradient represents logFC difference between contrast.

**Supplementary Figure S5. Inversed GS from the TFactS catalogue.**

a) Heatmap and hierarchical clustering of inversed GSs from the TFactS catalogue. Color corresponds to FC.

**Supplementary Tables**

**Supplementary Table 1. Primary antibodies list**

| **Antibody/Species** | **Company** | **Country/State** | **Reference** | **Method** | **Dilution** | **Blocking** |
| --- | --- | --- | --- | --- | --- | --- |
| ß-Actin ms | Sigma | United States, Massachusetts | A5316 | WB | 1:100,000 | 5% BSA in 0.1% TBST |
| ß-Catenin (CTNNB1) | Abcam | United Kingdom | Ab6302 | WB | 1:2000 | 5 % Milk in 0.1 % TBST |
| Collagen 1 ms | Sigma-Aldrich | United States, Massachusetts | c2456 | WB | 1:2000 | 5 % Milk in 0.1 % TBST |
| Collagen 2 rb | Thermo Fisher Scientific | United States, Massachusetts | BS-0709R | WB | 1:500 | 5 % Milk in 0.1 % TBST |
| Collagen 4 rb | Thermo Fisher Scientific | United States, Massachusetts | PA5-104508 | WB | 1:500 | 5 % Milk in 0.1 % TBST |
| Collagen 4 rb | Abcam | United Kingdom | ab6586 | IHC | 1:500 | 3 % BSA + 0.2 % Triton in PBS+/+ |
| CREB rb | Abcam | United Kingdom | ab32515 | WB | 1:500 | 5% BSA in 0.1% TBST |
| GTP-Tubulin human | AdipoGen Life Sciences | United States, California | AG-27B-0009-C100 | WB | 1:1000 | 5% BSA in 0.1% TBST |
| p-Kif20a rb | Boster | United States, California | A051 42S528-1 | IHC | 1:200 | 3 % BSA + 0.2 % Triton in PBS+/+ |
| NeuN gp | SYSY | Germany | 26604 | IHC | 1:1000 | 3 % BSA + 0.2 % Triton in PBS+/+ |
| NFKB rb | Cell Signalling Technology | United States, Massachusetts | cs 13586 | WB | 1:1000 | 5% BSA in 0.1% TBST |
| P-CREB (Ser) rb | Abcam | United Kingdom | ab32096 | WB/ICC | 1:5000 | 5% Milk in 0.1% TBST |
| PLK1 rb | Proteintech | Singapore | 10305-1-AP | ICC | 1:200 | 3 % BSA + 0.2 % Triton in PBS+/+ |
| Tubulin ms | Invitrogen/ Thermo Fisher Scientific | United States, Massachusetts | TF 32-2600 | WB | 1:1000 | 5% BSA in 0.1% TBST |

**Supplementary Table 2. Secondary antibodies list**

| **Antibody** | **Company** | **Country/State** | **Reference** | **Dilution** | **Blocking** |
| --- | --- | --- | --- | --- | --- |
| Mouse Anti-Human IgG2Rd HRP Conjugate | Invitrogen/ Thermo Fisher Scientific | United States, Massachusetts | MH-1722 | 1:1000 | 5% BSA in 0.1% TBST |
| Polyclonal rabbit-anti mouse Immunoglobin/HRP | Agilent Technology | United States, California | P0260 | 1:3000 | As used for respective primary antibody |
| Polyclonal goat-anti rabbit Immunoglobin/HRP | Agilent Technology | United States, California | P0448 | 1:1000 | As used for respective primary antibody |
| AlexaFlour® 488 donkey anti-rabbit | Jackson Immuno research | United Kingdom | 711-545-152 | 1:500 | 3 % BSA + 0.2 % Triton in PBS+/+ |
| AlexaFlour® 594 donkey anti-rabbit | Jackson Immuno research | United Kingdom | 711-585-152 | 1:500 | 3 % BSA + 0.2 % Triton in PBS+/+ |
| AlexaFlour® 647 donkey anti-rabbit | Jackson Immuno research | United Kingdom | 711-605-152 | 1:500 | 3 % BSA + 0.2 % Triton in PBS+/+ |
| AlexaFlour® 488 donkey anti-mouse | Jackson Immuno research | United Kingdom | 715-545-151 | 1:500 | 3 % BSA + 0.2 % Triton in PBS+/+ |
| AlexaFlour® 594 donkey anti-mouse | Jackson Immuno research | United Kingdom | 715-585-151 | 1:500 | 3 % BSA + 0.2 % Triton in PBS+/+ |
| AlexaFlour® 647 donkey anti-mouse | Jackson Immuno research | United Kingdom | 715-605-151 | 1:500 | 3 % BSA + 0.2 % Triton in PBS+/+ |
| AlexaFlour® 488 donkey anti-chicken | Jackson Immuno research | United Kingdom | 703-545-155 | 1:500 | 3 % BSA + 0.2 % Triton in PBS+/+ |
| AlexaFlour® 594 donkey anti-chicken | Jackson Immuno research | United Kingdom | 703-585-155 | 1:500 | 3 % BSA + 0.2 % Triton in PBS+/+ |
| AlexaFlour® 647 donkey anti-chicken | Jackson Immuno research | United Kingdom | 703-605-155 | 1:500 | 3 % BSA + 0.2 % Triton in PBS+/+ |
| AlexaFlour® 488 donkey anti-gp | Jackson Immuno research | United Kingdom | 705-545-147 | 1:500 | 3 % BSA + 0.2 % Triton in PBS+/+ |
| AlexaFlour® 594 donkey anti-gp | Jackson Immuno research | United Kingdom | 705-585-147 | 1:500 | 3 % BSA + 0.2 % Triton in PBS+/+ |
